# Design Principles of Biological Oscillators through Optimization: Forward and Reverse Analysis

**DOI:** 10.1371/journal.pone.0166867

**Published:** 2016-12-15

**Authors:** Irene Otero-Muras, Julio R. Banga

**Affiliations:** BioProcess Engineering Group, IIM-CSIC, Spanish National Research Council, Vigo, Spain; Spanish National Research Council (CSIC), SPAIN

## Abstract

From cyanobacteria to human, sustained oscillations coordinate important biological functions. Although much has been learned concerning the sophisticated molecular mechanisms underlying biological oscillators, design principles linking structure and functional behavior are not yet fully understood. Here we explore design principles of biological oscillators from a multiobjective optimization perspective, taking into account the trade-offs between conflicting performance goals or demands. We develop a comprehensive tool for automated design of oscillators, based on multicriteria global optimization that allows two modes: (i) the automatic design (forward problem) and (ii) the inference of design principles (reverse analysis problem). From the perspective of synthetic biology, the forward mode allows the solution of design problems that mimic some of the desirable properties appearing in natural oscillators. The reverse analysis mode facilitates a systematic exploration of the design space based on Pareto optimality concepts. The method is illustrated with two case studies: the automatic design of synthetic oscillators from a library of biological parts, and the exploration of design principles in 3-gene oscillatory systems.

## Introduction

Sustained oscillatory behavior can be generated by a simple negative feedback loop in combination with a time delay [1]. Biological oscillators, however, usually show a more complex structure. Mammalian circadian rhythms, for example, include multiple (negative and positive) feedback and feedforward loops in their underlying transcriptional networks [2]. Other significant oscillators like the sino-atrial node (mammalian heart’s natural pacemaker) and the cell cycle oscillator rely on circuits containing both positive and negative feedback loops [3]. The reasons for this complexity are in many cases not fully understood, and many efforts are devoted to identify design principles underlying the complex architectures selected through evolution (either organism- or function-specific properties or design principles shared by different organisms and functions).

Quoting Goldbeter [4]: *“In view of the large number of variables involved and of the complexity of feedback processes that generate oscillations, mathematical models and numerical simulations are needed to fully grasp the molecular mechanisms and functions of biological rhythms.”* In fact, mathematical models and computational approaches have already helped to build synthetic oscillators [5–8] (we will refer here to this design problem as forward analysis). Similarly, they have been used to identify underlying design principles [3, 4, 9–18] (we will refer here to this problem as reverse analysis).

By means of quantitative modeling, Tyson and Novak [14] demonstrated four general requirements (structural and parametric) in biological oscillators: negative feedback, time delay, sufficient ‘nonlinearity’ of the reaction kinetics and proper balancing of the timescales of opposing chemical reactions.

In a computational study, Tsai et al [3] found that, in circuits with both negative and positive feedbacks, a higher positive feedback strength led to a better capacity to adapt the period to cell demands. This property, referred to as period tunability, is found advantageous in a wide range of biological oscillators including the cell cycle.

In this work, we propose a multiobjective optimization-based design approach to the analysis of biological oscillators, suitable for both forward and reverse analysis.

Why optimization-based design? Optimization provides a **systematic and efficient** manner to explore the potential selective pressure over a particular feature of a biological oscillator (by considering the circuit realization as the outcome of an optimization-based design procedure). In this way, it is possible for example to investigate what environmental conditions drive specific oscillatory network architectures [19, 20], or to find core oscillatory modules with specific properties, for example minimal numbers of nonlinearities and components [15, 21]. In a more broader context, optimization strategies are being successfully applied to gene regulatory circuit design [22, 23].

Why a multiobjective approach? We assume that certain structural and parametric characteristics of biological oscillators could be explained by the fact that they are subject to **trade-offs between conflicting goals or demands**. Rand et al [24] explored the relationships between various desirable properties of circadian rhythms, postulated as *evolutionary aims*, and suggested a relation between complexity of the circuit and degree of flexibility (understood as the number of desirable properties that can be tuned simultaneously). While some of the evolutionary aims for circadian clocks are independent, other properties were found to be in a trade-off, as it is the case for entrainability to synchronize with external stimuli and regularity to oscillate with a precise period [1]. Multicriteria (Pareto) optimality concepts are being increasingly used to analyze/design complex systems in different contexts [25–27], including RNA design [28], bacterial adaptability [29, 30], metabolic networks [31–35], gene regulation [23, 36] and biosystems engineering [37].

Following the analysis of Tsai et al [3] of a model of the cell cycle, in which the period tunability (improved by increasing the positive feedback strength) was postulated as an evolutionary aim, we try to mimic the evolutionary process by an optimization procedure, in which the tunability of the period is an objective to maximize. A single optimization problem (without any further constraint) can lead to unrealistic values of the positive feedback strength. Intuitively, the stability of the oscillator appears as a biologically meaningful opposing objective to take into account. In this work we find that period tunability and stability of the oscillation are in a trade-off, and considering both as opposing objectives to maximize in a multiobjective problem gives as a result realistic values of the feedback strength.

Our optimization-based design approach relies on Mixed-Integer Nonlinear Programming (MINLP) methods, which provide computational efficiency to handle the required levels of complexity [38, 39]. The approach is described in the Methods section, addressing in detail (i) how to formulate the design of an (oscillatory) gene regulatory network as a multiobjective mixed-integer dynamic optimization problem, including potential objective functions to be selected, and (ii) how to solve efficiently the multiobjective problem to obtain the Pareto front.

In the Results section, we apply our methodology for both forward and reverse analysis of oscillators.

**Fig 1:**
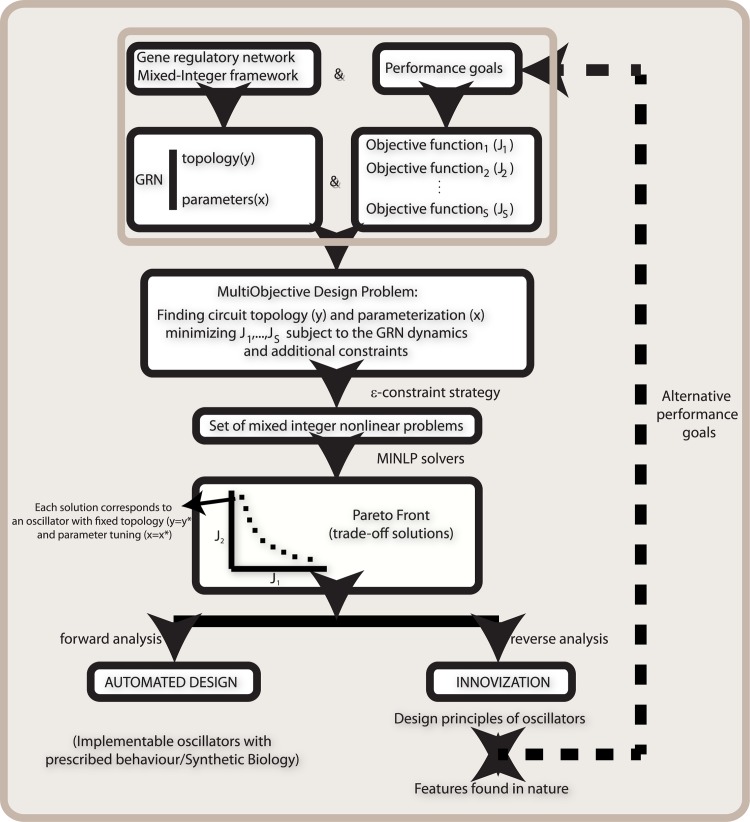
Workflow scheme of the Mixed-Integer multiobjective oscillator automated design process.

Within the above mentioned optimization-based framework, the forward analysis problem (left branch in the workflow in Fig 1) consists of systematically finding circuits capable of sustained oscillations (and also optimizing additional performance goals) among all the circuits that can be obtained by combining components of a given database (or library) of biological parts. Additional design criteria taking into account implementation issues can also be added.

Also within the optimization-based framework, the reverse analysis problem (right branch in the workflow in Fig 1) consists of finding structural/parametric patterns which allow us to infer design principles of biological oscillators. First we perform an optimization-based search through the topology-parameter spaces, aiming to find circuits (topology and parameters) leading to oscillations. Second, we proceed to extract innovative design principles through analysis of the optimization results, similarly to what have been recently called innovization procedures in engineering [40]. In other words, reverse analysis allows us to systematically uncover design principles from sets of optimal trade-off (Pareto) solutions.

## Methods

### Multiobjective Mixed-Integer Design Framework

Optimization-based design aims to find the design (or designs) with the best overall performance (in this case sustained oscillations) among the set of all possible circuits (search space). In this work we focus on oscillations at the transcriptional-translational level, and our approach is based on dynamic models of gene regulatory networks.

We employ a **Mixed-Integer description** of the gene regulatory network dynamics that is not constrained to a particular kinetics or model granularity. On the contrary, the description is generic and relies on the following assumptions:

1. A circuit in the search space is completely characterized by a vector of integer (and/or binary) variables (accounting for the topology or configuration) and a vector of real variables (accounting for parameter values).
2. the dynamics of the gene regulatory network can be encoded in a system of Ordinary Differential Equations (ODEs) of the form:
  $$\begin{array}{c} \dot{z}\left(t\right)=f\left(z,y,x,k\right),\;z\left(0\right)=z_{0} \end{array}$$ (1)

where:

- $z\in R^{N}$ is the vector of dynamic state variables coding for the levels of all the species involved in the circuit (we will denote its time derivative by $\dot{z}$);
- $x\in R^{R}$ is the vector of continuous decision variables containing the tunable parameters;
- $y\in Z^{M}$ is the vector of integer (or binary) decision variables determining the circuit model structure;
- $k\in R^{K}$ is a vector of fixed parameters.

In what follows *N* refers to the number of states (levels of the species involved) of the model, *R* is the number of tunable continuous parameters, *M* is the number of integer or binary variables defining the circuit structure and *K* is the number of fixed parameters. A number of gene regulatory network examples (with different kinetics and levels of detail) are included in the S1 Appendix.

Within this framework, a design goal can be encoded in an objective function of the form $J(\dot{z},z,x,y,k)$ such that the predefined behavior (design target) is achieved when *J* reaches its minimal value. Multiple design criteria are defined through a vector of objective functions $J=(J_{1},J_{2},\ldots ,J_{S})$.

We formulate the automated design of a gene regulatory network as finding a vector $x\in R^{R}$ of continuous variables and a vector $y\in Z^{M}$ of integer variables which minimize the vector $J=(J_{1},J_{2},\ldots ,J_{S})$ of objective functions:

$$\begin{array}{c} \min_{x,y}\;\;J_{1}\left(\dot{z},z,x,y,k\right),J_{2}\left(\dot{z},z,x,y,k\right),\ldots ,J_{S}\left(\dot{z},z,x,y,k\right) \end{array}$$ (2a)

subject to:

1. the circuit dynamics in the form of ODEs or Differential Algebraic Equations (DAEs) with the state variables *z* and additional parameters *k*:
  $$\begin{array}{c} \xi \left(\dot{z},z,x,y,k\right)=0,\;\;z\left(t_{0}\right)=z_{0}, \end{array}$$ (2b)
2. additional requirements (performance specifications and/or physicochemical limitations) in the form of equality and inequality constraints:
  $$\begin{array}{c} h(z,x,y,k)=0, \end{array}$$ (2c)
  $$\begin{array}{c} g(z,x,y,k)\leq 0, \end{array}$$ (2d)
3. upper and lower bounds for the real and integer decision variables:
  $$\begin{array}{c} x_{L}\;\leq \;x\;\leq \;x_{U}, \end{array}$$ (2e)
  $$\begin{array}{c} y_{L}\;\leq \;y\;\leq \;y_{U}. \end{array}$$ (2f)

The solution of the Multiobjective Optimization (MOO) problem consists of a set of points denoted as Pareto optimal [41, 42], and the set of all Pareto optimal solutions is known as Pareto front. A feasible circuit defined by (x*, y*) is a Pareto optimal solution of the multiobjective optimization problem if it is not dominated by other feasible circuits. Given two pairs (x*, y*), (x**, y**), we say that *J*(x*, y*) dominates *J*(x**, y**) if *J*(x*, y*) ≤ *J*(x**, y**) for all *J*_*i*_ (i=1,…,S) with at least one strict inequality.

For those readers not familiar with multiobjective optimization note that the above is a vector optimization problem [41]. In a multiobjective optimization problem, the utopia (or ideal) point is the one that optimizes all objective functions simultaneously as if they were considered in isolation (see the S1 Appendix). The utopia point is unattainable if at least two objectives are in contrast with each other, since optimizing one of the objectives will damage the others.

In terms of theoretical optimality all the solutions in the Pareto front are equivalent. In engineering design, the so called decision maker needs to define posterior preferences and evaluate them along the Pareto frontier in order to choose the best solution for implementation. In absence of posterior preferences, a common practice is to select the solution closest to the utopia point (this compromise solution is usually called the knee point). In this work, we adopt this additional selection criterion, and in what follows, we refer to the circuit with minimum distance to the utopia point as the circuit with best performance (in the context of multiobjective optimization).

### Design objectives for sustained oscillations in gene regulation

The single-objective optimization-based design of an oscillator requires to define an objective function whose minimization results in the desired oscillatory response. We introduce an objective function based on the autocorrelation of time series, which we prove to be well suited and effective for the search of sustained oscillators.

For a multiobjective design approach, we consider the sustained oscillatory behavior as requirement (constraint), and propose the tunability of the period and the stability of the limit cycle stability as criteria to be optimized. After introducing the autocorrelation function, we will provide mathematic definitions for both performance objectives and justify their selection as design targets.

#### Autocorrelation Function

A number of objective functions for oscillatory behavior can be found in the literature, based on fits to oscillatory dynamics or Fourier transforms [15]. Here we make use of the autocorrelation function.

Let *s*_*t*_ be a time series corresponding to a process which is ergodic and stationary. The autocorrelation function of *s*_*t*_ is defined as:

$$\begin{array}{c} \Gamma \left(t\right)=\left\langle s\left(t\right)s\left(0\right)\right\rangle =\lim_{T\to \infty }\frac{1}{T}\int_{0}^{T}d\tau s\left(t+\tau \right)s\left(\tau \right) \end{array}$$ (3)

We normalize this function to get Γ_*norm*_(*t*) = Γ(*t*)/Γ(0) such that the maximum value is Γ_*norm*_(0) = 1.

For *s*_*t*_ being the output of a deterministic simulation with sustained oscillatory behavior, the autocorrelation function Eq (3) oscillates in a sustained manner, and the first peak in the normalized autocorrelation function, in what follows denoted by *P*_*norm*Γ_, takes its maximum value 1.

If *s*_*t*_ describes a realization of a stochastic oscillatory process, i.e., it corresponds to the number of molecules *Z*^*i*^(*t*) of a species *i*, the autocorrelation Γ(*t*) shows a damped oscillation, due to the fact that stochastic fluctuations induce the phase diffusion of the oscillator and affect its periodicity [10]. The height of the first peak of the autocorrelation gives a measure of the precision of the stochastic oscillator [43]. The precision of the oscillators is usually quantified through the so-called quality factor, defined as *Q* = 2*πγ*/*T* where *γ* is the inverse of the damping rate or characteristic time of the decay of the autocorrelation function [16] and *T* is the period of the oscillation [10]. The quality factor *Q* is an estimation of the number of oscillations over which the periodicity is maintained [44] (note that higher *P*_*norm*Γ_ results in better *Q*). The quality factor is directly related also to the so called dissipation constant of the oscillator [45].

Therefore, we select −*P*_*norm*Γ_ as the objective to minimize in searching for oscillatory circuits. By minimizing this function we maximize the oscillator’s precision in case of stochastic time series and ensure a perfect (non damped) oscillation in case of deterministic dynamics when the objective function reaches its minimum value (-1).

In addition to its efficacy in the search for oscillators, the objective function chosen has additional advantages in terms of biological insight.

Taking *s*(*t*) = *Z*^*i*^(*t*) in Eq (3), the time average is equal (in stationary processes) to an average over the stationary probability distribution for the initial molecular number *Z*^*i*^(0), and we can establish a relation with the solution of the Chemical Master Equation (CME) as it has been derived by [10].

On the other hand, a relation between the dissipation constant of the oscillator (directly computable from the envelope of the autocorrelation function) and the free energy dissipated in one cycle of the oscillator has been found in a recent work by [45], leading to the conclusion that cells consume energy to improve the precision of the oscillator (robustness against intrinsic molecular noise). This result supports our selection of the first peak of the autocorrelation function as a biologically meaningful objective to optimize.

#### Tunability of the period

**Fig 2:**
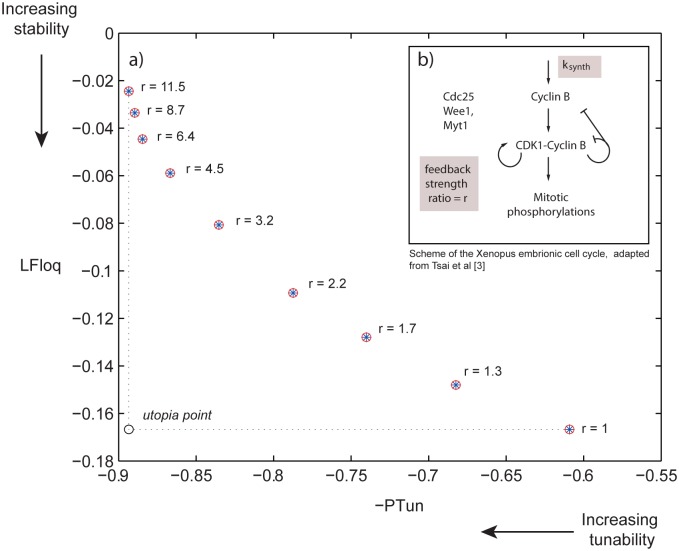
Leading Floquet Exponent plotted versus (negative) of the Period Tunability for the model of the mitotic oscillator. There is a trade-off between both properties for the model of the mitotic oscillator in [46] (*r* denotes here the positive feedback strength).

A wide range of biological oscillators, from the cell cycle to the sino-atrial node oscillator, require to adjust their frequency to the organism’s demands without compromising the amplitude of the oscillations. Starting from a model of the mitotic oscillator, Tsai et al [3] found that the tunability of the period (understood as the variability of the period without compromising the amplitude of the oscillations) increased with the positive feedback strength, indicating that circuits containing positive feedback might have been selected through evolution in cases where tunable frequency is desired. The period of the oscillator was changed by varying the rate constant for cyclin B synthesis, *k*_*synth*_ (see Fig 2b).

Here, we define the period tunability *PTun* with respect to an input or manipulable variable range *θ* as follows:

$$\begin{array}{c} PTun=T_{max}\left(\theta \right)-T_{min}\left(\theta \right) \end{array}$$ (4)

where *T*_*max*_(*θ*) and *T*_*min*_(*θ*) are the maximum and minimum values of the period within the manipulable variable interval. For convenience, we can normalize the function dividing by a constant in order to obtain a maximum at *PTun* = 1. The maximum variation of the amplitude allowed can be considered through an additional constraint.

Using the improved model for the mitotic oscillator in Xenopus by Tsai et al [46], we evaluate, as in the original work by [3], the tunability of the period with respect to the cyclin B synthesis rate constant *k*_*synth*_. If we consider a single-criterion optimization process where the unique objective is to maximize the period tunability, we obtain unrealistically high values of the feedback strength. Therefore we introduce (at least) another (potential) evolutionary aim exerting pressure in the opposing direction to obtain, as an outcome of an optimization process, more realistic values of the feedback.

#### Stability of the limit cycle

In order to test a potentially conflicting design objective we introduce here the stability of the limit cycle, evaluated through Floquet analysis. The stability of the limit cycle is here understood as the robustness of the oscillator against perturbations of the trajectory.

Let us consider the dynamics of an oscillator with period *T* to be described by Eq (1). The Jacobian of the system, $D_{z}f=\frac{\partial f}{\partial z}$, is a continuous *T*-periodic *N* × *N* matrix function. We define a linear matrix first order initial value problem:

$$\begin{array}{c} \dot{\Phi }=D_{z}f\Phi ,\;\;\Phi \left(0\right)=Id \end{array}$$

where *Id* is the *N* × *N* identity matrix. The monodromy matrix of the system is computed as:

$$\begin{array}{c} \Phi \left(T\right)=\int_{0}^{T}D_{z}f\Phi dt,\;\;\Phi \left(0\right)=Id. \end{array}$$

the eigenvalues of the monodromy matrix are the so-called Floquet multipliers:

$$\begin{array}{c} Floq_{m}=eig\left(\Phi \left(T\right)\right). \end{array}$$

Floquet multipliers are dimensionless numbers that give the period-to-period increase/decrease of a small perturbation away from the limit cycle. There is a multiplier equal to 1, corresponding to perturbations along the direction of the cycle, and the moduli of the remaining multipliers determine the stability of the limit cycle. The Floquet exponents:

$$\begin{array}{c} Floq=\ln\frac{|Floq_{m}|}{T}, \end{array}$$

have rate units *time*^−1^ and describe the mean contraction/expansion rate per one period of the orbit.

If any Floquet multiplier has a modulus greater than one (equivalently a Floquet exponent has a positive real part) the perturbation increases along the corresponding direction and the limit cycle is unstable.

For stable limit cycles, the leading Floquet exponent (denoted in what follows as *LFloq*) is an indication of how fast the system returns to its stable original periodic orbit after a perturbation. This has been found to correlate with the robustness of the oscillator against molecular noise, in the stochastic version of the model [47].

Using again the improved model for the mitotic oscillator in Xenopus by Tsai et al [46], we evaluate the Floquet exponents, and we find that performance indices, *LFloq* and *PTun*, are in a trade-off, as it can be deduced from the Pareto front in Fig 2. Importantly, the feedback strength decreases as we move along the Pareto front (from lower to higher stability of the limit cycle).

Therefore, the selection of *LFloq* and *PTun* as objective functions is justified based on: i) the two variables appear to be in a trade-off in the model of the mitotic cell cycle oscillator, ii) the corresponding multiobjective problem leads to realistic values of the positive feedback strength (similar to those found in nature).

A direct relation between the leading Floquet exponent and the envelop of the autocorrelation function has been established by [48], showing that the leading Floquet exponent gives a measure of the robustness of the oscillator with respect to molecular (intrinsic) noise. In this way, by optimizing the leading Floquet exponent, we are using a deterministic measure (computed from the deterministic ODE description of the oscillator) to optimize robustness against molecular noise without the need of stochastic simulations.

### Computing the Pareto front

Computing the Pareto optimal set is a challenging task in the context of biological circuits where search spaces can be large and combine real and integer variables, and the expected Pareto front might be discrete and/or non-convex, due to the high nonlinearity and the presence of integer variables.

Many methods have been developed to solve MOO problems. A typical classification [41] is based on the role of the decision maker, and includes (i) no-preference methods, (ii) a posteriori methods, (iii) a priori methods and (iv) interactive methods. We select the *ε*-constraint method, which belongs to the category of a posteriori methods and it is based on scalarization techniques, i.e. conversion of the original MOO problem to a set of single-objective optimization problems (in our case MINLP problems). In contrast to goal attainment method (which is an a priori method) the *ε*-constraint method does not require the pre-definition of reference goals [41], a major advantage in biosystems engineering applications, where in general such references are unknown.

The proposed optimization process is composed of the following steps, considering two objective functions *J*_1_ and *J*_2_:

1. Search for the optima of each of the individual objectives:
  $$\begin{array}{c} \left(x_{1}^{*},y_{1}^{*}\right),\;\;\left(x_{2}^{*},y_{2}^{*}\right). \end{array}$$
2. Compute the individual objective bounds as:
  $$\begin{array}{c} \underline{J_{1}}=J_{1}\left(x_{1}^{*},y_{1}^{*}\right),\;\bar{J_{1}}=J_{1}\left(x_{2}^{*},y_{2}^{*}\right), \end{array}$$
  $$\begin{array}{c} \underline{J_{2}}=J_{2}\left(x_{2}^{*},y_{2}^{*}\right),\;\bar{J_{2}}=J_{2}\left(x_{1}^{*},y_{1}^{*}\right). \end{array}$$
3. Select the objective function to be minimized, denoted in what follows as the primary objective (without loss of generality let us take *J*_1_ as the primary objective).
4. For the non-minimized objective *J*_2_, generate a vector
  $$\begin{array}{c} \varepsilon =[\varepsilon_{1},\ldots ,\varepsilon_{i},\ldots ,\varepsilon_{m}] \end{array}$$
  such that $\varepsilon_{1}\leq {\underline{J}}_{2}$, $\varepsilon_{m}\geq {\bar{J}}_{2}$ and $\varepsilon_{1}<\varepsilon_{2}<\ldots <\varepsilon_{m}$.
5. Solve the MINLP:
  $$\begin{array}{c} \min_{w,y}\;\;J_{1}\left(\dot{z},z,x,y,k\right) \end{array}$$
  subject to:
  $$\begin{array}{c} \varepsilon_{k}\leq J_{2}\left(\dot{z},z,x,y,k\right)<\varepsilon_{k+1} \end{array}$$
  for k=1,…,m-1 by means of a MINLP solver.
6. Evaluate the solutions obtained and construct the Pareto front with the non dominated optimal ones.

The *ε*-constraint methodology described has two important advantages in the context of gene regulatory oscillators: all Pareto optimal solutions can be found (even for discrete and non-convex Pareto fronts) and, in addition, it allows to exploit the advantages of hybrid MINLP solvers.

Hybrid MINLP solvers combine global optimization metaheuristics with efficient local search methods, taking elements of both stochastic and deterministic optimization approaches. In [37], hybrid solvers have been shown to outperform pure evolutionary methods [49] in a number of Nonlinear Programming problems (real variables), since hybrid solvers required less function evaluations. In a previous work, we proved the efficiency of hybrid MINLP approaches for the design of gene regulatory networks [38]. Here we make use of three MINLP hybrid solvers that combine stochastic global search with the local Mixed-Integer Sequential Quadratic Programming (MISQP) by [50], namely the Enhanced Scatter Search algorithm (eSS) by [51], the Mixed-Integer Tabu Search algorithm (MITS) by [52] and the Mixed-Integer Ant Colony Optimization (ACOmi) by [53].

## Results

### Forward analysis: automated design of oscillators from a library of biological parts

In the context of forward analysis the modeling framework needs to ensure modularity and easy translation of the model into an implementable circuit. The use of standard parts allows to transfer experimental data to mathematical models and facilitates the design of gene regulatory systems [22].

We follow the formalism from the Registry of Standard Biological Parts [54] and consider the following basic constitutive components of genetic circuits: *promoters* recruiting the transcriptional machinery which transcribes the downstream DNA sequence, *ribosome binding sites* controlling the accuracy and efficiency with which the translation of mRNA begins, *protein coding regions* containing the sequence information needed to create a functional protein chain and *terminators* signaling the end of transcription.

**Fig 3:**
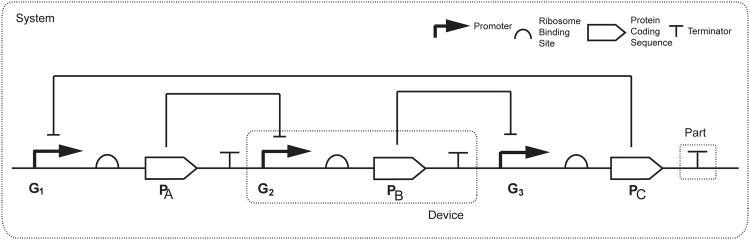
Repressilator regulatory system [5]. The system consists of three genes connected in a feedback loop. The first gene in the circuit expresses some protein A which represses the second gene, the second gene expresses a protein B which represses the third gene, and protein C expressed by the third gene closes the feedback loop by repressing the first gene.

The abstraction hierarchy proposed by Endy [54] classifies standard parts in three different layers: *parts*, defined as sequences with basic biological functions (like for example DNA binding proteins), *devices* (combinations of parts with a particular function) and *systems* (combinations of devices). This is illustrated in Fig 3 through the Repressilator regulatory system [5], where the different devices and parts are indicated.

We start from a library of biological parts, where each part in the library is endowed with a set of reactions. The full set of reactions for a given circuit is obtained from the reactions of its constitutive parts. Regarding the reactions associated with each part type, we adopt the formalism proposed by Pedersen and Phillips [55], where, for a device where the promoter G is repressed by a protein P, the following reactions are considered:

1 Binding of the repressor G+P→GP,
2 Unbinding of the repressor GP→G+P,
3 Transcription GP→GP+mP,
4 Translation mP→mP+P,
5 Protein degradation P→∅.

All the reactions are endowed with mass action kinetics, and the dynamics of the all the species (including mRNA) are taken into account (see the S1 Appendix for details). It is important to remark that within this formalism, the set of reactions associated to each part is easy to extend in order to consider e.g. hybrid promoters, different degrees of cooperativity, promoters controlling multiple transcription factors, etc). Here we extend the original set of reactions to incorporate:

6 Degradation of bound repressor GP→G.

To accommodate the dynamics into our Mixed-Integer description, let us denote by *G* the number of promoters, *B* the number of ribosome binding sites, *P* the number of protein coding regions and *A* the number of terminators in the library of biological parts. The number of possible device configurations (in what follows we refer specifically to protein generator devices) is *M* = *G* × *B* × *P* × *A*. We label every possible device with an integer index i=1,…,M and build a vector $y\in Z^{M}$ of binary variables such that:

$$\begin{array}{c} \left\{ \begin{array}{c} y_{i}=1\;,\;\text{if}\;\text{the}\;\text{device}\;i\;\text{is}\;\text{part}\;\text{of}\;\text{the}\;\text{circuit}\;\text{structure},\\ y_{i}=0\;,\;\text{otherwise}. \end{array}\right. \end{array}$$

The structure of a gene regulatory circuit is completely defined by the vector *y*.

Fixed kinetic parameters are collected in a vector $k\in R^{K}$, whereas manipulable parameters are contained in the vector $x\in R^{R}$. Importantly, we can select any parameter to be tuned (for example the strength of the RBS). In some design problems, it might be of interest to select an external inducer as a decision variable. In this case, the external inducer will be part of the “tunable parameters” in the problem formulation.

The dynamics are given by Eq (1), with:

$$\begin{array}{c} f(z,y,x,k)=N(y)v(x,y,k)\; \end{array}$$ (5)

where *N*(*y*) is the stoichiometric matrix and *v*(*x*, *y*, *k*) is the vector of rates of the reaction network (depending nonlinearly on the species in accordance with the mass action law).

A database of biological parts, adapted from Pedersen and Phillips [55] has been coded in Matlab. The Matlab library contains 4 promoters: $G_{1}=P_{\lambda }$, $G_{2}=P_{tet}$, $G_{3}=P_{bad}$, $G_{4}=P_{lac}$, 1 ribosome binding site, 1 terminator and 11 protein coding regions for the proteins *cIR*, *tetR*, *araC*, *lacI*, *luxI*, *luxR*, *lasR*, *lasI*, *ccdB*, *ccdA*, *ccdA*2. This makes a total of 44 possible devices, where each device contains a pair promoter-protein coding region, 1 ribosome binding site and 1 terminator. Labeling each of this devices with a number from 1 to 44, the structure of a circuit is completely defined by a vector *y* with 44 binary entries. Note that (without additional constraints on the number of devices) circuits can contain from 1 to 44 different devices. The nominal values of the kinetic parameters are taken from [55].

For a given pair (*y*, *x*), the model equations of the corresponding gene network are automatically generated.

We can impose a maximum number of devices (*D*_*max*_) in the solution circuit(s) by setting:

$$\begin{array}{c} \sum_{i=1}^{M}y_{i}\leq D_{max} \end{array}$$

First, we solve a single objective design problem aiming to find endogenous oscillators among the combinations of devices in the library, minimizing −*P*_*norm*Γ_. The number of possible different devices (binary decision variables for the optimization based design) is *n* = 44 and we set a maximum of three devices *D*_*max*_ = 3. We use in first instance the original version of the library (without degradation of bound repressor).

*Stochastic regime.* The constraints imposed by the dynamics are obtained here by simulation with the stochastic Gillespie algorithm [56] (the kinetic constants are adjusted accordingly). In this way, we are taking into account the effect of intrinsic noise [57], i.e. stochastic fluctuations associated with intracellular reactions. In order to tackle extrinsic sources of noise (due to unequal partition of cellular material at cell division), mathematical frameworks like Stochastic Variable Number Monte Carlo by [58] should be used.

**Fig 4:**
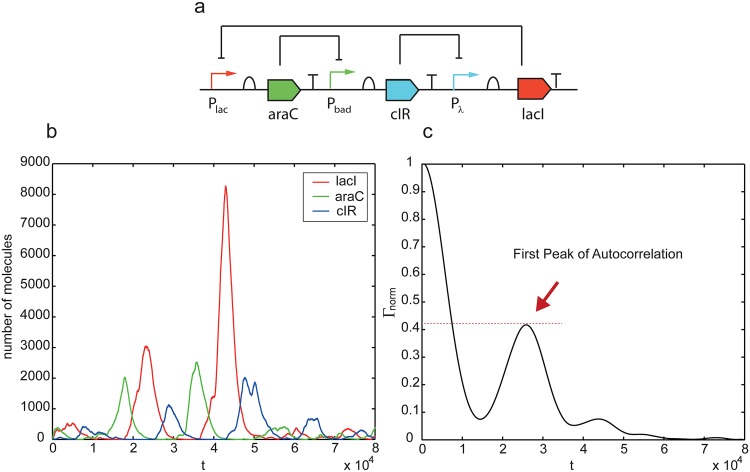
Stochastic realization and autocorrelation function for the best oscillator found by the algorithm from the library of biological parts (*D*_*max*_ = 3).

The best oscillator found consists of the three devices *P*_*lac*_-*rbs*-*araC*-*ter*, *P*_*bad*_-*rbs*-*cIR*-*ter*, *P*_*λ*_-*rbs*-*LacI*-*ter* following the Repressilator configuration. The circuit is depicted in Fig 4, together with the dynamics obtained by the Gillespie algorithm for a single realization and the autocorrelation function for the *cIR* stochastic dynamics.

*Deterministic regime.* In the deterministic regime, no circuit was found leading to sustained oscillatory behavior. We initially employ a multistart strategy (20 runs of 600 seconds from different random initial guesses) using eSS, MITS and ACOmi, and after increasing the number of runs and computation times arrived to the same result.

**Fig 5:**
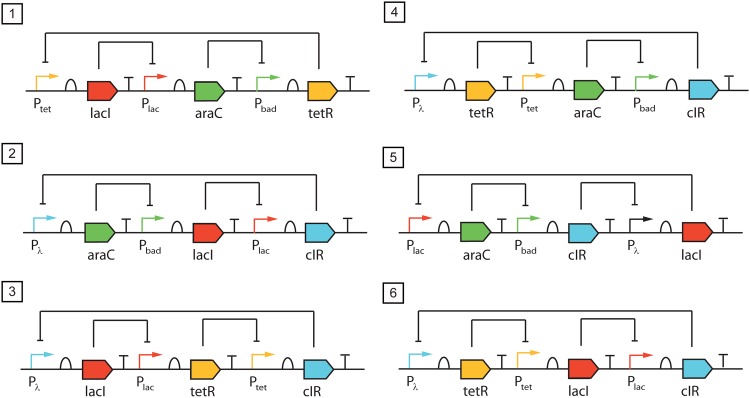
Deterministic oscillators found by the algorithm from the augmented library of biological parts (taking into account the degradation of the bound repressor) with *D*_*max*_ = 3. Circuit 3 corresponds to the original Repressilator system by Elowitz [5].

We further use the extended library including the degradation of bound repressor. With the same multistart strategy we found six different circuits, all of them endowed with the Repressilator topology illustrated in Fig 3 where $P_{A}$ represses $G_{2}$, $P_{B}$ represses $G_{3}$, and $P_{C}$ represses $G_{1}$. We include the solutions in Fig 5.

The six circuits perform optimally with respect to the single objective (sustained oscillations). Additional criteria are needed in order to select the best circuit for further implementation. Next, we compute the values of the leading Floquet exponent *LFloq* (as indicated in Eq (1)) and the Period tunability *PTun* (understood here as the variation of the period with respect to the protein degradation constant) according to Eq (4). The values obtained are shown in Table 1. Only three of the circuits (corresponding to structures 1, 4 and 5 in Fig 5) are found to be Pareto optimal with respect to these two design criteria.

**Table 1:** Values of the period tunability (normalized) and leading Floquet exponent for the circuits in Fig 5.

	Circuit 1	Circuit 2	Circuit 3	Circuit 4	Circuit 5	Circuit 6
PTun	0.9758	0.9400	0.8147	0.9560	0.9428	0.8147
LFloq	−0.0017	−0.0016	−0.0018	−0.0024	−0.0025	−0.0015

We evaluate now the distance to the utopia point from each of these solutions, which is found to be minimal for the circuit 4.

**Fig 6:**
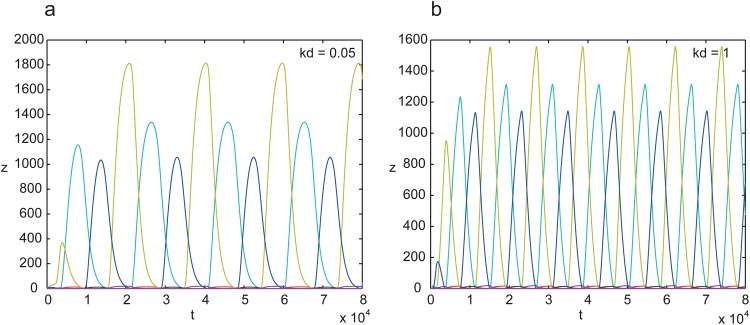
Dynamics of circuit 4, for low (a) and high (b) values of the degradation constant *kd*. It can be observed that the frequency of the oscillator is higher for the circuit with higher *kd*.

In Fig 6, the dynamics of circuit 4 for low and high values of the degradation constant are depicted.

Importantly, note also that the circuit with highest leading Floquet exponent, i.e. circuit 5, is the one which was previously found to be more robust with respect to molecular noise in the stochastic framework (maximizing the first peak of the autocorrelation function).

In summary:

1. Six different structures leading to sustained oscillations where found in the deterministic regime, taking into account the degradation of the bound repressor. All the oscillatory circuits found are endowed with a Repressilator-type structure (the original Repressilator is included among the solutions, corresponding to circuit 3 in Fig 5).
2. No deterministic oscillators were found from combinations of parts in the library, without taking into account the degradation of the bound repressor.
3. In the stochastic regime, oscillators are found without taking into account the degradation of the bound repressor. These results are coherent with [59], where oscillatory behavior is precluded in absence of cooperativity and without degradation of the bound repressor for the Repressilator, and it is shown that deterministic and stochastic methods might not agree about the existence of oscillations.
4. Using as criterion the shortest distance to the utopia point, we selected the Repressilator-type circuit with the best performance with respect to both stability of the limit cycle and tunability of the period.
5. The oscillator with highest limit cycle’s stability (highest leading Floquet multiplier) in the deterministic regime is found to be the most robust with respect to molecular noise in the stochastic regime.

### Reverse analysis: uncovering design principles of oscillatory gene regulatory networks

In the context of reverse analysis we use a species-based representation, biologically-verified and extensively employed in the study of developmental gene networks [60, 61], in which a circuit is defined by the signs and strengths of the interactions. Within this framework, a *N*-gene regulatory network is described by a directed graph where the nodes are genes and the edges indicate their interactions (one arrow from gene A to gene B indicates the transcriptional regulation of B by the transcription factor encoded by A). The regulation from gene $G_{i}$ to gene $G_{j}$ is characterised by two numbers:

- an integer *y*_*ij*_ ∈ {−1, 0, 1}, coding for inhibition (−1), no action (0), and activation (1);
- a strictly positive weight $x_{ij}\in R_{>0}$.

The gene-gene interaction indices and the weights are contained in two matrices $Y\in Z^{N\times N}$ and $X\in R_{>0}^{N\times N}$ respectively (where *N* is the number of genes in the network). The effective regulating input to a gene $G_{i}$ is given by:

$$\begin{array}{c} \chi_{i}=\sum_{j=1}^{N}\omega_{ji}z_{j}+\alpha_{i}I \end{array}$$ (6)

where *ω*_*ji*_ = *y*_*ji*_ *x*_*ji*_, and the term *α*_*i*_ *I* reflects the effect of external inputs (in case the gene $G_{i}$ is only affected by internal gene-gene interactions, the coefficient *α*_*i*_ = 0). The transcription rate is proportional to the sigmoidal-filtering of the total contribution, such that the balance for the protein *z*_*i*_ encoded by $G_{i}$ reads:

$$\begin{array}{c} {\dot{z}}_{i}=\frac{1}{1+exp(a-b\left(\chi_{i}\right))}-\delta z_{i} \end{array}$$ (7)

where parameters *a* and *b* control the steepness and location of the threshold value of the regulation function, and *δ* is the protein degradation rate constant. The formalism complies with the requirements *A*.1 and *A*.2 in **Methods** section with a vector $x\in R_{>0}^{R}$ containing the weights (its elements are taken column-wise from *X*), and a vector of binary variables $y\in Z^{M}$ determining the interactions (its elements are taken column-wise from *Y*). Note that *R* = *M*. Parameters *a*, *b*, *δ* and other fixed parameters are included in a vector $k\in R^{K}$.

In previous studies, Tyson and Novak [62] reported two different 3-gene motifs with capacity for oscillatory behavior: the negative feedback loop motif, and the amplified negative feedback loop motif. Besides, Kim et al [63] found that coupled negative-negative feedback loops enforce oscillatory behavior. Exploring the dynamics of basic signalling modules, Kholodenko [64] reported 32 different positive-negative feedback designs with capacity for oscillations (for some of them, a degree of cooperativity of the feedback regulations is required for oscillatory behavior).

Here, we are interested in evaluating whether feedforward loops can produce, in combination with additional connections, sustained oscillations.

**Fig 7:**
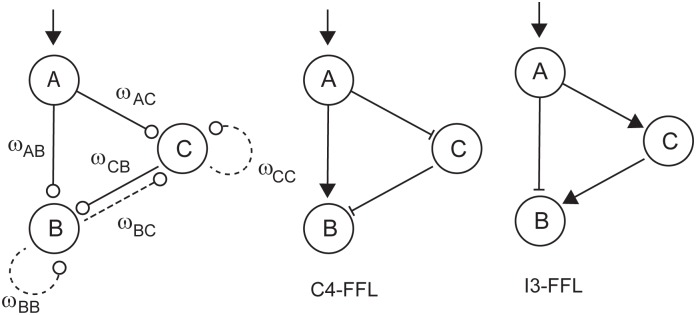
Superstructure for the 3-gene feedforward motif with additional connections (adapted from [61]). Middle and right structures correspond to the coherent feedforward motif C4-FFL and the incoherent feedforward motif I3-FFL.

We consider the 3-gene network in Fig 7 with genes A, B and C, where the net internal interaction matrix is given by:

$$\begin{array}{c} \Omega =(\begin{array}{ccc} 0 & 0 & 0\\ \omega_{AB} & \omega_{BB} & \omega_{CB}\\ \omega_{AC} & \omega_{BC} & \omega_{CC} \end{array}) \end{array}$$

and the gene A is induced by an external input *I*. The ODE system describing the dynamics of this network, according to Eqs (6) and (7) reads:

$$\begin{array}{ccc} \dot{A} & = & \frac{1}{1+exp(a-b(I))}-\delta A\\ \dot{B} & = & \frac{1}{1+exp(a-b(\omega_{AB}A+\omega_{BB}B+\omega_{CB}C))}-\delta B\\ \dot{C} & = & \frac{1}{1+exp(a-b(\omega_{AC}A+\omega_{BC}B+\omega_{CC}C))}-\delta C. \end{array}$$ (8)

*A*, *B* and *C* denote the levels of species A, B and C. The binary variables *y*_*AB*_, *y*_*AC*_ and *y*_*CB*_ determine what we denote as underlying feedforward configuration of the circuit, whereas *y*_*BB*_, *y*_*BC*_ and *y*_*CC*_ define additional interactions of *B* and *C* self-activation (or deactivation) and mutual inhibition-activation from *B* to *C*. The values of the input, parameters and initial conditions are included in the S1 Appendix.

There are eight possible underlying feedforward structures (with active *AB*, *AC* and *CB* connections), corresponding to the four coherent (C) and four incoherent (I) feedforward (FFL) motifs [65]. In Fig 7 the structures for C4-FFL and I3-FFL motifs are depicted.

**Fig 8:**
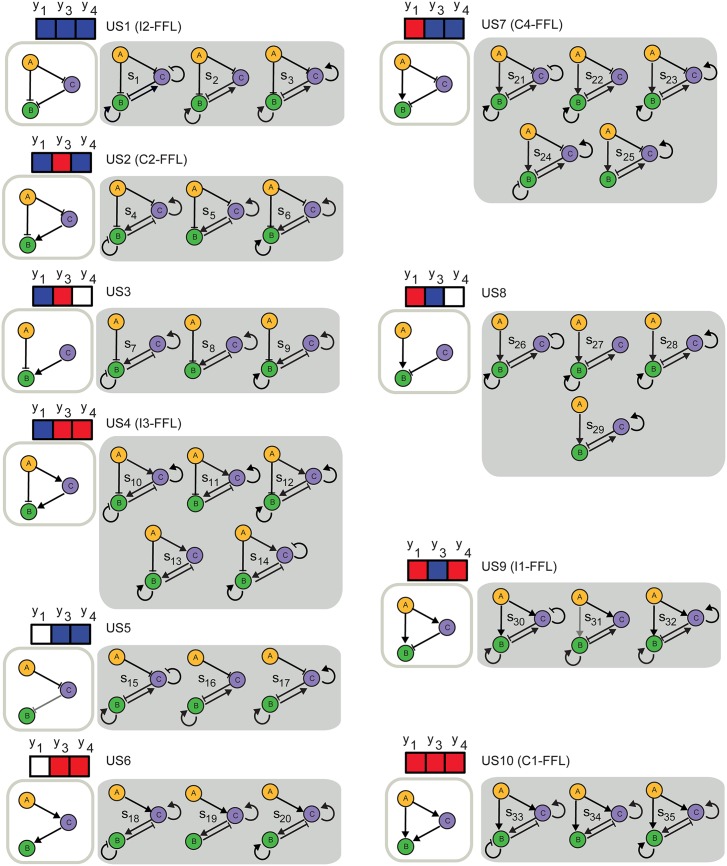
3-gene topologies (all of them correspond to regulated feedback motifs) leading to oscillatory behavior (structures *s*_1_ to *s*_35_), grouped in terms of the corresponding underlying feedforward loop type. Underlying feedforward topologies (US1 to US10) are defined by the values of the integer variables *y*_1_, *y*_3_ and *y*_4_. Red, blue and white entries represent positive (+1), negative (-1) and absence of regulation (0), respectively. All of them include a regulated feedback motif, as defined by [65].

First, we formulate a single objective design problem minimizing *J*_1_ = −*P*_*norm*Γ_ to find oscillatory circuits. There are six integer variables *y*_1_ = *y*_*AB*_, *y*_2_ = *y*_*BB*_, *y*_3_ = *y*_*CB*_, *y*_4_ = *y*_*AC*_, *y*_5_ = *y*_*BC*_ *y*_6_ = *y*_*CC*_ describing the sign of the connections and six real variables *x*_1_ = *x*_*AB*_, *x*_2_ = *x*_*BB*_, *x*_3_ = *x*_*CB*_, *x*_4_ = *x*_*AC*_, *x*_5_ = *x*_*BC*_ and *x*_6_ = *x*_*CC*_ describing the strengths. We follow a multistart strategy similar to the previous application, finding 35 different circuit structures leading to oscillations, depicted in Fig 8.

We can classify the obtained oscillatory topologies in ten different groups, according to the values of the integer variables *y*_1_ = *y*_*AB*_, *y*_3_ = *y*_*CB*_ and *y*_4_ = *y*_*AC*_ which define the feedforward loop type. Ten different underlying topologies (denoted by US1 to US10 in Fig 8) are found. Among them, we find three coherent feedforward motifs: C1-FFL, C2-FFL and C4-FFL (corresponding to US10, US2 and US7 in Fig 8), three incoherent feedforward motifs: I1-FFL, I2-FFL and I3-FFL (corresponding to US9, US1 and US4 in Fig 8) and four degenerated structures (lacking one of the principal connections), corresponding to US3, US4, US6 and US8 in Fig 8.

**Fig 9:**
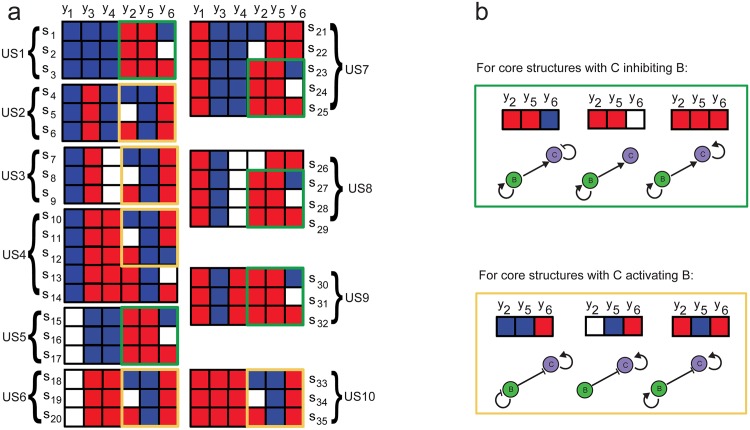
3-gene topologies leading to oscillatory behaviour. a) Schemes for the 3-gene topologies producing sustained oscillations. Red, blue and white entries represent positive (+1), negative (-1) and absence of regulation (0), respectively. b) Recurrent sets of additional interactions are enclosed by yellow and green rectangles.

In order to look for recurrent additional-connection patterns among the circuits found we use the diagram in Fig 9a, in which the connections are represented by colors.

It can be observed that no oscillatory circuit is found without any active additional connection (*y*_2_ = *y*_*BB*_, *y*_5_ = *y*_*BC*_, *y*_6_ = *y*_*CC*_) (feedforward loops alone have not the capacity to create oscillations). Actually, two additional connections are necessarily active (negative or positive) in all oscillators. Importantly, we find recurrent patterns in connections (*y*_2_ = *y*_*BB*_, *y*_5_ = *y*_*BC*_, *y*_6_ = *y*_*CC*_) leading to oscillators. The three combinations depicted in Fig 9b (green) appear always in oscillatory circuits with a negative value of *y*_3_ = *y*_*CB*_ in its underlying FFL structure, while the combinations in Fig 9b (yellow) appear always in oscillatory circuits with a positive value of *y*_3_ = *y*_*CB*_ in its underlying FFL structure. According to these results, a negative feedback between *B* and *C* genes is needed for an oscillation showing that activation-repression cores embedded within feedforward loops produce oscillations. Most of the feedforward structures require also of *B* self-activation (in case of negative *y*_3_ = *y*_*CB*_), or *C* self-activation (in case of positive *y*_3_ = *y*_*CB*_), except for C4-FFL and I3-FFL where oscillations appear also without this additional requirement.

Importantly, all the structures found include a regulated feedback motif as defined by [65], since an activation-repression negative feedback between *C* and *B* (in which the activator is amplified by self-activation or by the upstream transcription factor) is always present. Note that this core feedback leads to symmetries between circuits with core topologies US1-US2, US3-US5, US4-US7, US6-US8 and US9-US10.

Next we formulate a multiobjective problem setting as design objectives the leading Floquet exponent *LFloq* (as indicated in **Methods** section) and the Period tunability *PTun* (understood here as the variation of the period with respect to the input) according to Eq (4). Here, the condition for oscillations is set as an additional inequality constraint where the first peak of the autocorrelation function is greater than a predefined threshold *P*_*norm*Γ_ > *P*_*norm*Γ__*thr*_.

**Fig 10:**
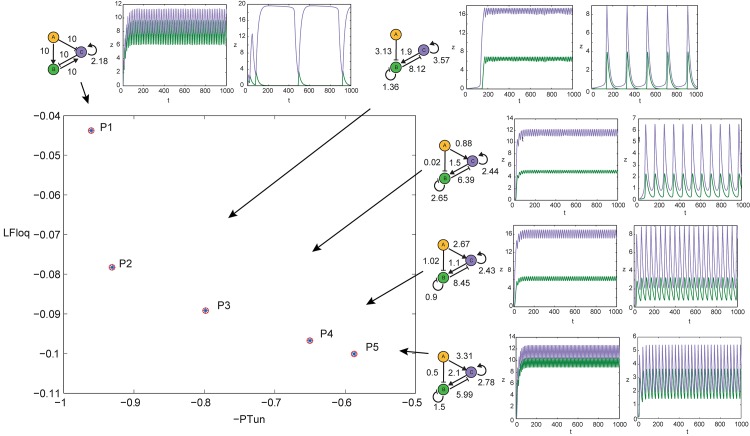
Pareto Front of 3-gene motifs with underlying feed-forward structure showing a trade-off between period tunability and stability of the limit cycle. The structure and parameters for each circuit indicated, together with the dynamics of the oscillator for the lower and upper values of the input *I*. Circuit P1 exhibit a significantly greater period tunability (oscillations at lower and upper values of the input have very different periods) than the other circuits in the Pareto Front.

Using the *ε*-constraint strategy described in **Methods** section, we obtain a set of non-dominated points depicted in Fig 10, where it can be observed that only two different underlying feedforward structures appear in the Pareto front: the coherent feedforward motif 4 (C4-FFL) and the incoherent feedforward motif (I3-FFL). The first point in the Pareto Front (maximum value of the period tunability) corresponds to a C4-FFL circuit. There is an intermediate point (P2) with no effect of gene A on gene C. As the value of the tunability decreases, the topology changes to I3-FFL. We observe that the strength of the *y*_*AC*_ connection increases as we move along the Pareto front.

In summary:

1. Ten different (proper or degenerate) feedforward structures (all of them with an embedded enhanced negative feedback caused by an activation-repression core) are found leading to oscillatory behavior. Importantly, all the oscillatory structures contain a regulated negative feedback motif (as defined by [65] and no feedforward circuit alone (without additional connections) is found to be capable of sustained oscillations.
2. Activation-repression cores embedded within feedforward loops produce sustained oscillations (we find that negative feedback between *B* and *C* genes is necessary for oscillatory behavior). Most feedforward structures require also of *B* self-activation (in case of negative *y*_3_ = *y*_*CB*_), or *C* self-activation (in case of positive *y*_3_ = *y*_*CB*_), except for C4-FFL and I3-FFL. Recurrent additional-connection patterns are summarized in Fig 9.
3. A set of non-dominated circuits are found to show an optimal trade-off between period tunability and stability of the limit cycle.
4. Activation-repression cores embedded within feedforward loops C4-FFL and I3-FFL produce optimal oscillators in terms of robustness and period tunability.
5. The oscillator topology evolves in a structured manner along the Pareto Front, changing from (activation-repression) cores embedded within a C4-FFL loop to cores embedded within a I3-FFL structure as tunability decreases and robustness (limit cycle stability) increases.

## Conclusions

We developed a global mixed-integer optimization approach for the analysis of biological oscillators. This approach is valid for deterministic and stochastic description of the dynamics, makes use of the autocorrelation function to detect sustained oscillatory behavior, and allows the incorporation of multiple design criteria. We illustrated how this approach is useful for both forward engineering and reverse analysis of biological oscillators.

We propose the first peak of the autocorrelation function as an objective to maximize in stochastic oscillators, which allows for effective search of oscillators with optimal robustness with respect to molecular (intrinsic) noise. Oscillator robustness with respect to parameters has been analyzed in a recent paper by [66]. In the deterministic regime, we propose the leading Floquet multiplier as an objective to optimize, in order to find oscillators with optimal attractivity of the limit cycle. We have also shown through an example that, in accordance with relations previously established between autocorrelation and Floquet multipliers [48], optimizing the leading Floquet in the deterministic regime provides optimal robustness with respect to intrinsic noise in the stochastic regime. A recent work by [45] shows a relation between the phase diffusion constant of an stochastic oscillator and the free-energy dissipation per cycle, indicating that cells may consume energy (ATP) in order to maintain the coherence of oscillations. This supports the selection of both objectives (first peak of the autocorrelation in stochastic regime and Floquet in deterministic regime) as meaningful potential evolutionary aims in the context of biological oscillators. The opposing objective chosen in this work (tunability of the period), has been already postulated as an evolutionary aim by [3] for a wide range of oscillators including the cell cycle. We found that tunability of the oscillator and stability of the limit cycle are in trade-off for the mitotic cell cycle oscillator [3], in which the existence of more than one design objective was needed to obtain, as the outcome of an optimization procedure, realistic values of the feedback strength.

*Forward engineering of biological oscillators (automated design):* single objective design problems can have several (possibly infinite) solutions with similar performance, where no extra information is obtained to select the best circuit for implementation. On the contrary, introducing multiple opposing objectives lead to well-defined design problems, where the solution is a Pareto front of non-dominated points, ordered by increasing/decreasing values of each of the objective functions (this aspect has been illustrated in [38]). We propose the distance to the so-called utopia point as a criterion to select the best oscillator for implementation.

Starting from a library of biological parts, we searched for circuits capable of endogenous sustained oscillations. Without taking into account degradation of bound repressors, oscillators where found only in the stochastic regime. After extending the library to introduce degradation of the bound repressor, we found circuits capable of sustained oscillations in both stochastic and deterministic regimes. Using the extended library, we found six different structures (of the Repressilator type) leading to sustained oscillators. Taking into account as additional design criteria the period tunability and the stability of the limit cycle, the original Repressilator is not recovered as an optimal one in the Pareto Front. This is not strange, as the original Repressilator design is not based on optimization (in particular, it has not been designed to optimize any of the criteria we are taking into account). By means of a multiobjective optimization formulation, we found other Repressilator-type structures performing better than the original Repressilator in terms of period tunability and limit cycle stability.

The multiobjective approach can be useful to implement circuits with the ability to mimic some of the desirable properties appearing in natural oscillators. For example, in the design of synthetic circadian clocks [67], where the oscillator needs to satisfy at least: persistence under constant conditions (precise period), entrainability by light/dark signals and temperature compensation of the period [68].

*Reverse analysis of biological oscillators (exploring design principles):* the existence of trade-offs among opposing performance goals might be important to explain the circuit complexity found in natural oscillators.

We searched for circuits (topology and parameters) giving rise to sustained oscillators in a 3-gene feedforward superstructure, leading to interesting observations. First, all the structures found to oscillate include a regulated negative feedback consisting in an activation-repression core embedded within the feedforward loop. Regulated feedbacks (a two node feedback is regulated by an upstream transcription factor) are, according to [65] a family of motifs or patterns of interconnections occur in natural transcriptional networks at frequencies much higher than those found in randomized networks. In particular, regulated feedbacks are found to be over-represented in developmental transcription networks.

Performing a multiobjective (tunability vs stability) design we observed that activation-repression cores embedded within feedforward loops C4-FFL and I3-FFL produce optimal oscillators (fulfill the trade-off relationship between period tunability and stability of the limit cycle). Moreover, the oscillator topology evolves in a structured manner along the Pareto Front, changing from an activator-repressor core embedded into a C4-FFL to an activator-repressor core embedded into a I3-FFL, as tunability decreases and limit cycle stability increases.

We propose a multiobjective iterative procedure to systematically explore design principles of biological oscillators: starting from a vector of design objectives, compute the set of non-dominated solutions and infer a set of patterns or design principles from the Pareto front. Then, compare the obtained patterns with the architectures found in natural oscillators. In case of divergence, a new set of objective functions is considered, and the Pareto front of solutions updated, in an iterative process. Any property of interest in the design of oscillators, and/or postulated as a potential evolutionary aim can be encoded as an objective in the design problem, including protein production cost, robustness against variability in the protein levels, period entrainability with an external signal. For the case of stochastic oscillators, the mean period and the precision have been also suggested as evolutionary aims [16]. The selection of the design objectives depends on *a priori* advantageous properties for the case under study. These advantageous properties can be radically different, for example, between the cell cycle oscillators and a circadian clock [1, 69, 70].

## Supporting Information

S1 Appendix Pdf file containing additional information. (PDF)

S1 File Zip file containing Matlab code. (ZIP)
