# Supplementary material for: Design Principles of Biological Oscillators through Optimization: Forward and Reverse Analysis
Source: PLoS One. 2016 Dec 15;11(12):e0166867. doi: 10.1371/journal.pone.0166867 (PMC5158198; doi:10.1371/journal.pone.0166867)
Supplement: S1 Appendix — (PDF) [file pone.0166867.s001.pdf]

Design Principles of Biological Oscillators through optimization: Forward and Reverse Analysis Supporting Information: S1 Appendix

I. Otero-Muras and J. R. Banga

October 16, 2016

Contents

1 **Shape of the Pareto Front in the performance space** **2**

2 **Encoding dynamics of gene regulation in a mixed-integer description** **4**

3 **Case Study 1 additional information** **6**

4 **Case Study 2 additional information** **10**

Chapter 1

Shape of the Pareto Front in the performance space

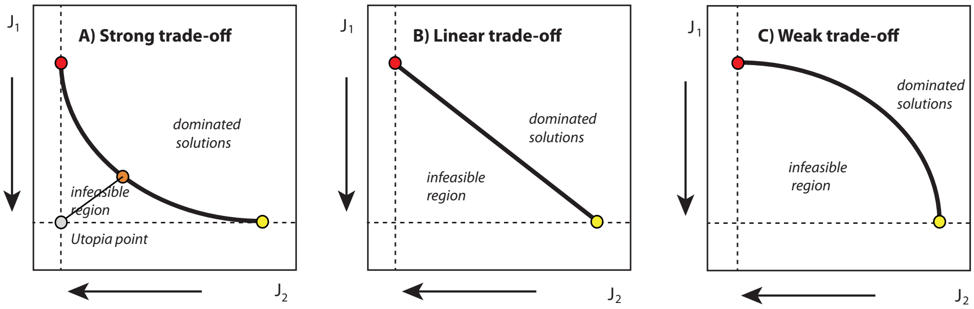

Figure S1: Pareto front examples in a two dimensional performance space. A) Convex Pareto front (strong trade-off), B) Linear Pareto front (linear trade-off), C) Concave Pareto front (weak trade-off). The extremes of the Pareto front are indicated by red and yellow dots. The utopia point (ideal point in general unattainable) is defined as the point of extreme (best) values for each objective function (obtained by minimizing each objective function independently).

Fig. S1 shows examples of Pareto fronts in a two dimensional performance space. In this approximate picture, using a continuous deterministic approach to modelling biological networks (in which no variability is considered among single cells), we consider that two features selected as competing evolutionary aims are expected to be in a trade-off, and evolved systems are expected to lie in the Pareto front.

Remark 1: Note that this picture is oversimplified (MINLP problems can

lead to discrete Pareto fronts with both convex and non convex regions).

Remark 2: Note that Fig. S1 corresponds to the case in which we are minimizing both objectives.

Chapter 2

Encoding dynamics of gene regulation in a mixed-integer description

As we state in the main text, the mixed integer framework we propose is not constrained to a particular kinetics or model granularity. This is shown next where we explain how a number of examples (with different kinetics and levels of detail) are accommodated into the generic mixed integer description.

Example 1: Hill kinetics, lumped transcription-translation. Starting from a set of promoter elements denoted by $\mathcal{G}_1, \ldots, \mathcal{G}_G$ and a set of transcript proteins $\mathcal{P}_1, \ldots, \mathcal{P}_P$, Dasika and Maranas [1] describe the dynamic model of the gene regulatory network by a set of ODEs of the form:

$$\dot{z}_j(t) = V_j(t) - K_{j_{decay}} z_j(t) \quad \forall j \tag{2.1}$$

where V_j is the generation/consumption rate of z_j due to the reactions and $K_{j_{decay}} z_j$ is the degradation rate. The rate expressions for the transcripts read:

$$V_j(t) = \sum_i Y_{ij} v_{ji}(t) \tag{2.2}$$

where v_{ji} is the rate of production of $\mathcal{P}_j$ from $\mathcal{G}_i$ (with kinetics of Hill type), and Y_{ij} is a binary variable such that:

$Y_{ij} = 1$ if production of protein $\mathcal{P}_j$ from promoter $\mathcal{G}_i$ is turned on

$Y_{ij} = 0$ otherwise.

In this way, the topology of the gene regulatory network is given by a super-structure $G \times P$ matrix Y containing the binary variables of the model (the

vector of binary variables $y \in \mathbb{Z}^M$ is obtained by converting the matrix Y to a vector by columns). Basal protein production and/or reactions among proteins and external inducers can be incorporated. The tunable parameters are contained in the vector of real variables $x \in \mathbb{Z}^R$.

Example 2: Mass action kinetics, detailed dynamics. Starting from a set of basic constitutive components of genetic circuits, including promoters, ribosome binding sites (RBSs) and protein coding regions, we can encode the dynamics of a circuit composed as a combination of parts from the library using a vector of binary variables $y \in \mathbb{Z}^M$, where M is the total number of devices that can be configured with the elements in the library, and the element y_i indicates whether the device is present in the circuit configuration ($y_i = 1$) or not ($y_i = 0$) [4].

In this way we adapt the reaction scheme proposed by [3] (with kinetics of mass action, mRNA dynamics being considered) to mixed integer framework, for the forward design of gene regulatory circuits (Case study 1 in the main text).

Example 3: Species-based representation. Simplified species graph-based representations are widely used to regulation among N genes (see for example [2]). Within this framework the regulation from gene $\mathcal{G}_i$ to gene $\mathcal{G}_j$ is characterised by two numbers, an integer number y_{ij} coding for the type of interaction, and a real number x_{ij} coding for the strength of the interaction. We use this approach for the reverse analysis of gene regulatory circuits (Case study 2 in the main text).

Chapter 3

Case Study 1 additional information

Here we include the standard part properties taken from [3] together with the extension proposed to incorporate the degradation of bound repressor. A promoter negatively regulated by a protein R has associated the reactions:

$$G + R \underset{k_u}{\overset{k_b}{\rightleftharpoons}} GR \xrightarrow{k_{tb}} GP + mP \tag{3.1}$$

where G is the promoter, R is the repressor protein, GR is the repressor-promoter complex and mP is the $mRNA$ of the transcribed protein. The parameters k_b, k_u and k_{tb} refer to the protein-promoter binding rate constant, protein-promoter unbinding rate constant and the rate of transcription in the bound state. The reactions corresponding to a promoter not regulated by any transcription factor are:

$$G \xrightarrow{k_t} G + mP \qquad mP \xrightarrow{k_{dm}} \emptyset \tag{3.2}$$

where k_t is the constitutive rate of transcription in absence of transcription factors and k_{dm} is the degradation rate constant for the $mRNA$ degradation. Here it is important to note that a promoter may show also positive regulation, multi-regulation either positively or negatively by the levels of multiple transcription factors, and both constitutive and regulated transcription. The ribosome binding site part has one associated reaction:

$$mP \xrightarrow{k_r} mP + P \tag{3.3}$$

where k_r is the rate constant corresponding to the translation of $mRNA$. Finally, the protein coding region part is endowed with:

$$P \xrightarrow{k_d} \emptyset \tag{3.4}$$

where k_d is the degradation rate constant of the protein P. Starting from a database of genetic parts with their respective relevant properties, one can obtain the complete reaction network for composed devices and systems. For example, the set of reactions for a device consisting of a promoter G_1, repressed by a protein P_1, and a ribosome binding site for the expression of a downstream protein P_2 will read, in presence of the repressor protein P_1 as:

$$G_1 + P_1 \underset{k_{u1}}{\overset{k_{b1}}{\rightleftharpoons}} G_1P_1 \xrightarrow{k_{tb1}} GP_1 + mP_2$$

$$mP_2 \xrightarrow{k_{r2}} mP_2 + P_2 \qquad P_2 \xrightarrow{k_{d2}} \emptyset \tag{3.5}$$

In order to consider the degradation of the bound repressor, we add the reaction:

$$GR \xrightarrow{k_{db}} G \tag{3.6}$$

to the previous scheme (3.1), where k_{db} represents the rate constant for degradation of the bound repressor.

In the following table we include the kinetic parameter values used in this work, taken from [3].

Table S1: Rates and values of the kinetic rate constants

rate	expression	k_i value
v_1	$k_1 \cdot P1 \cdot cIR$	1
v_2	$k_2 \cdot P1cIR$	0.5
v_3	$k_3 \cdot P1cIR$	0.00005
v_4	$k_4 \cdot P1$	0.12
v_5	$k_5 \cdot cIRm$	0.001
v_6	$k_6 \cdot cIRm$	0.1
v_7	$k_7 \cdot cIR$	0.001
v_8	$k_8 \cdot tetRm$	0.001
v_9	$k_9 \cdot tetRm$	0.1

v_{10}	$k_{10} \cdot tetR$	0.001
v_{11}	$k_{11} \cdot araCm$	0.001
v_{12}	$k_{12} \cdot araCm$	0.1
v_{13}	$k_{13} \cdot araC$	0.001
v_{14}	$k_{14} \cdot lacIm$	0.001
v_{15}	$k_{15} \cdot lacIm$	0.1
v_{16}	$k_{16} \cdot lacI$	0.001
v_{17}	$k_{17} \cdot luxIm$	0.001
v_{18}	$k_{18} \cdot luxIm$	0.1
v_{19}	$k_{19} \cdot luxI$	0.001
v_{20}	$k_{20} \cdot luxRm$	0.001
v_{21}	$k_{21} \cdot luxRm$	0.1
v_{22}	$k_{22} \cdot luxR$	0.001
v_{23}	$k_{23} \cdot lasRm$	0.001
v_{24}	$k_{24} \cdot lasRm$	0.1
v_{25}	$k_{25} \cdot lasR$	0.001
v_{26}	$k_{26} \cdot lasIm$	0.001
v_{27}	$k_{27} \cdot lasIm$	0.1
v_{28}	$k_{28} \cdot lasI$	0.001
v_{29}	$k_{29} \cdot ccdBm$	0.001
v_{30}	$k_{30} \cdot ccdBm$	0.1
v_{31}	$k_{31} \cdot ccdB$	0.001
v_{32}	$k_{32} \cdot ccdAm$	0.001
v_{33}	$k_{33} \cdot ccdAm$	0.1
v_{34}	$k_{34} \cdot ccdA$	0.1
v_{35}	$k_{35} \cdot ccdA2m$	0.001
v_{36}	$k_{36} \cdot ccdA2m$	0.1
v_{37}	$k_{37} \cdot ccdA2$	0.001
v_{38}	$k_{38} \cdot P2 \cdot tetR$	1
v_{39}	$k_{39} \cdot P2tetR$	0.5
v_{40}	$k_{40} \cdot P2tetR$	0.00005
v_{41}	$k_{41} \cdot P2$	0.09
v_{42}	$k_{42} \cdot P3 \cdot araC$	1
v_{43}	$k_{43} \cdot P3araC$	0.000001
v_{44}	$k_{44} \cdot P3araC$	0.00001
v_{45}	$k_{45} \cdot P3$	0.1
v_{46}	$k_{46} \cdot P4 \cdot lacI$	1
v_{47}	$k_{47} \cdot P4lacI$	0.5
v_{48}	$k_{48} \cdot P4lacI$	0.00005
v_{49}	$k_{49} \cdot P4$	0.1
v_{50}	$k_{50} \cdot P1cIR$	0.072
v_{51}	$k_{51} \cdot P2tetR$	0.072
v_{52}	$k_{52} \cdot P3araC$	0.072

v_{53}	$k_{53} \cdot P4lacI$	0.072

Chapter 4

Case Study 2 additional information

The next table contains the values of the parameters values for the connectionist model, taken from [2].

Table S1: Values of the parameters for the connectionist model

Parameter	Value
a	5
b	1
δ	0.05
I_L	Md^{c_L}
I_U	Md^{c_U}
M	5
d	0.982
c_L	30
c_L	1

Bibliography

[1] Dasika, M S and Maranas, C D (2008). OptCircuit: An optimization based method for computational design of genetic circuits *BMC Syst Biol* 2:24.

[2] Munteanu, A and Cotterell, J and Sole, RV and Sharpe, J (2014). Design principles of stripe-forming motifs:the role of positive feedback. *Sci Rep* 4:5003.

[3] Pedersen, M and Phillips, A (2009). Towards programming languages for genetic engineering of living cells. *J R Soc Interface* 6:S437–S450.

[4] Zomorrodi, A R and Maranas, C D (2014). Coarse-grained optimization-driven design and piecewise linear modeling of synthetic genetic circuits *Eur J Oper Res* 237:665–676.
